# Development of PZT Actuated Valveless Micropump

**DOI:** 10.3390/s18051302

**Published:** 2018-04-24

**Authors:** Fathima Rehana Munas, Gehan Melroy, Chamitha Bhagya Abeynayake, Hiniduma Liyanage Chathuranga, Ranjith Amarasinghe, Pubudu Kumarage, Van Thanh Dau, Dzung Viet Dao

**Affiliations:** 1 Faculty of Engineering, University of Moratuwa, Moratuwa 10400, Sri Lanka; v.roshal1992@gmail.com (G.M.); paychamitha@gmail.com (C.B.A.); hlchathuranga8@gmail.com (H.L.C.); ranamajp@gmail.com (R.A.); kumarap@uom.lk (P.K.); 2 Research Group (Environmental Health), Sumitomo Chemical, Hyogo 665-8555, Japan; dauthanhvan@gmail.com; 3 Queensland Micro- and Nanotechnology Centre, Griffith University, Gold Coast, Queensland 4215, Australia; d.dao@griffith.edu.au

**Keywords:** PZT, dual diaphragm, nozzle jet, microfluidics, micropump

## Abstract

A piezoelectrically actuated valveless micropump has been designed and developed. The principle components of this system are piezoelectrically actuated (PZT) metal diaphragms and a complete fluid flow system. The design of this pump mainly focuses on a cross junction, which is generated by a nozzle jet attached to a pump chamber and the intersection of two inlet channels and an outlet channel respectively. During each PZT diaphragm vibration cycle, the junction connecting the inlet and outlet channels with the nozzle jet permits consistencies in fluidic momentum and resistances in order to facilitate complete fluidic path throughout the system, in the absence of any physical valves. The entire micropump structure is fabricated as a plate-by-plate element of polymethyl methacrylate (PMMA) sheets and sandwiched to get required fluidic network as well as the overall device. In order to identify the flow characteristics, and to validate the test results with numerical simulation data, FEM analysis using ANSYS was carried out and an eigenfrequency analysis was performed to the PZT diaphragm using COMSOL Multiphysics. In addition, the control system of the pump was designed and developed to change the applied frequency to the piezoelectric diaphragms. The experimental data revealed that the maximum flow rate is 31.15 mL/min at a frequency of 100 Hz. Our proposed design is not only for a specific application but also useful in a wide range of biomedical applications.

## 1. Introduction

Onsite biomedical devices used in microfluidics are very popular and help to make human life healthy and comfortable. Though manufacturing these devices in a micro-scale is a great challenge and there is a growing demand. Among all the biomedical devices used in microfluidics applications, micropumps are essential [1,2,3,4,5,6,7]. Micropumps are defined as the diminished pumping elements fabricated by micromachining technologies. They are subcategorized as displacement micropumps and dynamic micropumps through the consideration of their driving fundamentals. In general, displacement micropumps are mechanically actuated and a physical actuator is essential in these kinds of pumps. On the other hand, dynamic micropumps are used in microdevices to increase the momentum of the working fluid by adding energy to it [2,3,7]. Hence, displacement micropumps are commonly used in biomedicine.

In micropumps, valve mechanisms are useful to control the fluid flow by opening, closing, and partially hindering passageways, therefore, the design of micropumps are subdivided into those with valves and those without valves (valveless). In micropumps with valves, an external actuation source is necessary for opening and closing the valves. Although monitoring the valves is comparatively easy, it is complicated within integrated microfluidic systems. In valveless micropumps, nozzle/diffuser elements are used as flow rectifiers. These elements work in such a manner that during supply mode more fluid enters through an inlet than the fluid exiting the outlet, and reverse action is directed during the pumping mode. 

Displacement-type valveless micropumps consist of a chamber closed by a flexible membrane, which is oscillated periodically by an actuation mechanism. This periodic movement of the membrane causes a periodic change in the fluid volume and pressure inside the pump chamber [2,8]. The flow directing elements attached to the pump chamber will act as inlet/outlet ports and direct the fluid from one element to another [2,3,4,9,10,11]. 

There are several micropumps designed and developed for different kinds of applications. The very first micropump was made in 1975 [3,12,13]. In 1988, a micropump with a valve was reported by van Lintel [1,2] and research and development studies in designing micropumps with various kinds of actuation principles continued. These micropumps were developed in different materials as well as with different micromachining techniques [2,3,14]. The very first valveless micropump was developed in 1993 by Stemme and Stemme [1,2,8]. The design and simulation of a polydimethylsiloxane (PDMS) membrane-based piezoelectric micropump was presented in 2016 [15]. 

Although from 1975 to 2016 there was research that continued the design and development of micropumps with different actuation techniques, many for biomedical applications were not user-friendly or portable. In addition, most of these devices were fabricated with silicon and were expensive. The developed devices lacked facilities to control the fluid flow according to user requirements.

In the present work, we developed a dual diaphragm, piezoelectrically actuated (PZT) valveless micropump using a simple fabrication technique to precisely control the fluid flow with polymethyl methacrylate (PMMA) sheets, in a cost-effective manner. 

## 2. Proposed Design and Working Principle of Micropump

**Figure 1:**
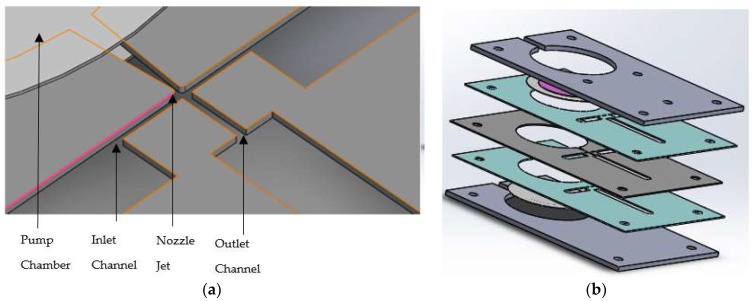
Details of designed model: (**a**) Details of the microfluidic channel; (**b**) Exploded view of the designed model.

The design of the micropump was mainly divided into two major components, which were the actuation mechanism and the fluidic network. The proposed design of the micropump system consisted of two identical 35 mm diameter with 0.3 mm thick circular PZT actuated metal diaphragms as actuators. The thickness of the PZT disk was 0.22 mm. The fluidic network was composed of a 30 mm diameter circular pump chamber enclosed with two PZT actuated membranes on the top and bottom, with one nozzle element, two inlet channels, and an outlet channel. Figure 1a expresses the details of these microfluidic channels. In order to allow smooth fluid flow through the channels and to reduce leakages and bubbles at the edges and walls of the micropump, the circular shaped designwas most preferable. In addition, compared with the other shapes of the membrane, circular diaphragms delivered better displacement for the same externally applied load [12,16,17]. The inlet and outlet ports were put on the top side of the micropump. 

As a rule of thumb, cross sections of the inlets should be larger than the outlet at the cross junction, in order to reduce fluidic resistance [18,19,20,21,22]. Hence, the designed cross sections of the inlet and outlet channels and nozzle jet were 0.5 × 1.5 (mm^2^), 0.6 × 0.5 (mm^2^) and 0.3 × 0.5 (mm^2^) respectively. The whole micropump structure was designed and fabricated as a plate-by-plate element of polymethyl methacrylate (PMMA) sheets and sandwiched to establish the required fluidic network. The exploded view of the model is expressed in Figure 1b. The whole packed size of the device was 100 mm × 60 mm × 5.5 mm. 

**Figure 2:**
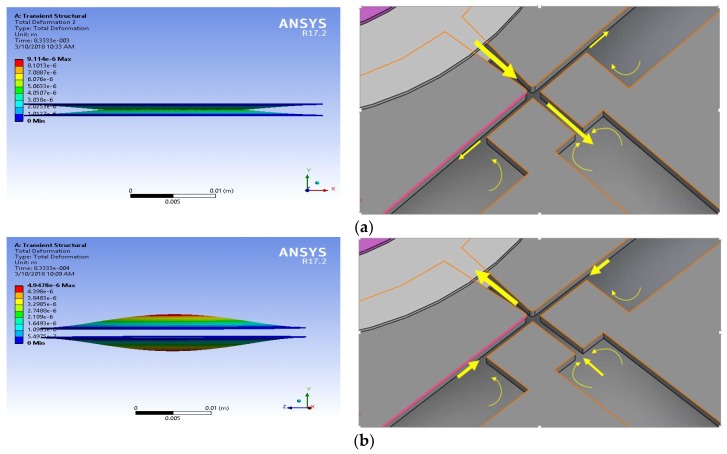
Illustration of working principle: (**a**) Deformation pattern of piezoelectrically actuated (PZT) diaphragm and the fluid flow motion during compression; (**b**) Deformation pattern of PZT diaphragm and the fluid flow motion during suction.

Based on deformations of the PZT actuated diaphragms, pumping action could be divided into two parts: suction and compression strokes. PZT diaphragms periodically deform in a concave and convex manner when PZT actuators were actuated with an alternating voltage. Thus, the volume of the chamber would vary periodically as expressed in Figure 2. In general, during the pumping stroke, PZT membranes were deflected in a convex pattern. Thus most of the fluid was directly expelled out through the outlet channel since inlet channels are perpendicular to the outlet (Figure 2a). On the other hand, during the suction stroke, PZT membranes were deflected in a concave pattern and the fluid is sucked into the chamber, due to the formation of low pressure inside. At this point, owing to the differences with forward momentum and fluid flow resistances along the channels at inlet/outlet, more fluid would directly enter through the inlet channels than the outlet (Figure 2b). 

## 3. Design and Simulation of Micropump

The analysis of the proposed micropump involved three fields of physics: structural mechanics, electrostatics, and fluid–structure interaction. The solutions of the piezoelectric structural and electrical analysis were explained by the piezoelectric strain–charge relationship. When a piezoelectric material is subjected to an electric field, the proportional mechanical strain is generated, and the linear piezoelectric constitutive strain–charge correlation for isothermal conditions using contracted matrix notation is represented by the following equations [17]:

(1) $$S_{ij}=S_{jk}^{E}T_{k}+d_{kj}E_{k}$$

(2) $$D_{i}=d_{ij}T_{j}+\in_{ij}^{T}E_{j}$$

*S* is the mechanical strain, *S^E^* is the elastic compliance coefficient at constant electric field, *T* is the mechanical stress, *d* is the piezoelectric strain coefficient, *D* is the electric displacement, *E* is the electrical field, ϵ*^T^* is the permittivity at constant stress, and *i*, *j* and *k* are vector notations along x, y, z directions respectively. 

In addition, the piezoelectric strain coefficient matrix for PZT-5H is given by:

(3) $$\left[d_{ij}\right]=\begin{array}{ccc} 0 & 0 & d_{31}\\ 0 & 0 & d_{31}\\ 0 & 0 & d_{33}\\ 0 & d_{15} & 0\\ d_{15} & 0 & 0\\ 0 & 0 & 0 \end{array}$$

*d*_31_ = −274 × 10^−12^ *C*/*N*, *d*_33_ = 593 × 10^−12^ *C*/*N*, *d*_15_ = 741 × 10^−12^ *C*/*N*. Thus, the residual stress is negligible and the term $S_{jk}^{E}T_{k}$ on the right side of Equation (1) goes to zero. In addition, it is assumed that the strain produced in a thin circular membrane is only in a radial direction. Therefore, the equation simplifies to:

(4) $$S_{1}=d_{31}E_{3}$$

**Figure 3:**
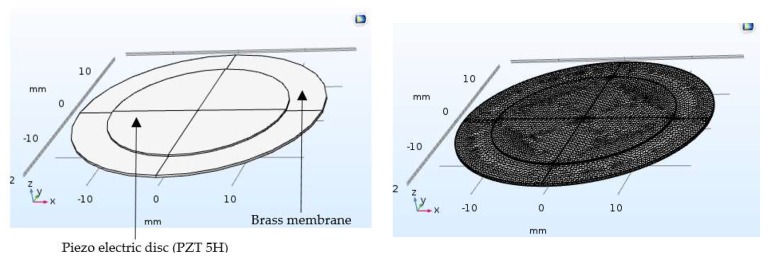
Designed and meshed models of the PZT actuated diaphragm.

In order to simplify the theoretical analysis, the piezoelectric structural and electrical simulation was executed in COMSOL Multiphysics 5.3 under the piezoelectric stress/strain mode on eigenfrequency study setup. The brass diaphragm was fixed around its perimeter, and the PZT 5H disk was bonded on top of the diaphragm. The characteristics of brass are expressed in Table 1. The air exposed model was meshed with the physics controlled mesh. The developed model and the meshed model are shown in Figure 3. Hence, the micropump diaphragm was deflected due to the PZT effect when applying an alternating electrical signal of 20 V. 

**Figure 4:**
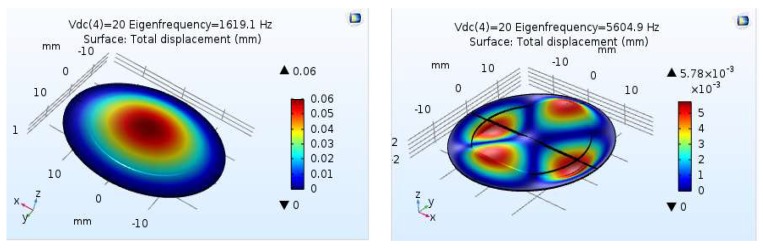
First and fourth modes of eigenfrequency analysis for piezoelectric PZT 5H diaphragm.

The structural analysis of the PZT 5H disc at the supplied voltages for different eigenfrequencies is shown in Figure 4. These results were useful in identifying the frequency at which maximum displacement occurred. This analysis revealed that the first eigenfrequency mode of 1619 Hz has the maximum center displacement of 0.06 mm. This was also the maximum displacement mode and gives the maximum volumetric volume change occurring frequency as well. This data would also be used to vary the frequency applied to the piezoelectric diaphragms of the micropump within this range. Therefore, the control system of the pump could be designed to apply an electric signal in which its frequency ranges from 1 Hz to 1619 Hz. On the other hand, in practice, the eigenfrequency values may vary during the piezoelectric disc fixing with the micropump.

The solution to the fluid flow inside the micropump is described by incompressible Navier–Stokes equations. The governing equations for the fluid motion are mass and momentum conservation and is described in the following manner with index notation [4,16,17,18,19,20,21,22,23,24,25,26].

(5) $$\nabla \cdot \vec{u}=0$$

(6) $$\rho \frac{\partial \vec{u}}{\partial t}+(\vec{u}\cdot \nabla )\rho \vec{u}=-\nabla p+\nabla \cdot (\eta \nabla \vec{u})$$

where, $\vec{u}$ and *p* are the velocity vector and pressure of the flow field; *ρ* and *η* are density and the dynamic viscosity of the flowing fluid respectively.

When the PZT actuators, which are bonded with the micropump diaphragms, were subjected to uniform force, the deflections of the diaphragms would be considered as the first symmetric motion mode of the thin circular plates which were uniformly fixed along its perimeter [17,20,21]:

(7) $$\varphi (r,t)=\Theta_{0}\sin(2\pi ft){\left[1-{\left(\frac{r}{R}\right)}^{2}\right]}^{2}$$

where *r*, *R* is a length measured from the center and diaphragm radius, *f* is the frequency of the load and $\Theta_{0}$ is the center amplitude of the solution.

It is assumed that the micropump works in the absence of preliminary pressure gradient. Also, negligible pressure was levied at the inlet and outlet and non-slip conditions were considered at the walls. Hence, motions of PZT diaphragms were transferred to the inlet velocities by time derivative of deflection, illustrated as follows [1,16,17,23].

(8) $$v(r,t)=(2\pi f\Theta_{0})\cos(2\pi ft){\left[1-{\left(\frac{r}{R}\right)}^{2}\right]}^{2}$$

During the transient structural simulation, the time step ∆*t* was selected in a manner where Δt=1/(fN). Here, N was selected as 12 to replicate the flow behavior throughout a single cycle of PZT diaphragm fluctuation.

**Figure 5:**
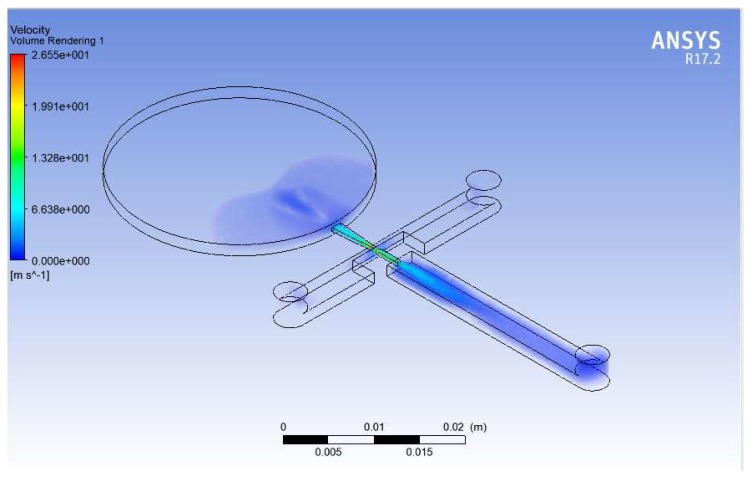
Volumetric plot of velocity profile.

**Figure 6:**
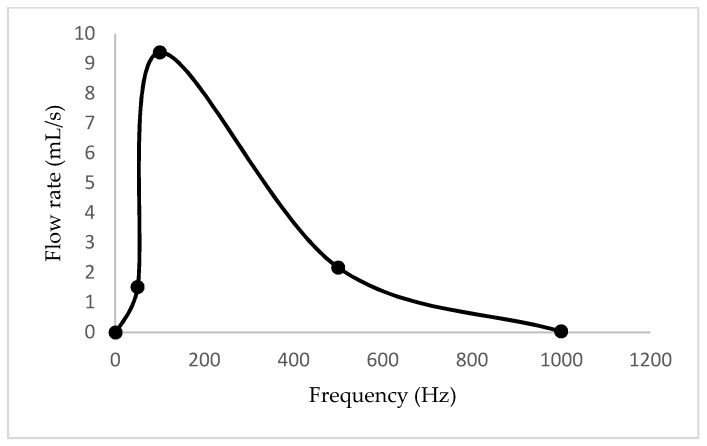
Variation of net flow rates with the frequency.

The solution of fluid flow described by the Navier–Stokes equations was obtained by coupled field analysis of ANSYS-Fluent. A second-order accuracy scheme was used for spatial derivatives and time advance approximations. The pressure-based transient solver method with the k-ε realizable model was used for the fluidic simulation. The SIMPLE scheme was considered for the velocity–pressure coupling algorithm. Liquid water at an ambient temperature was used as the working fluid. In addition, the fluidic part was meshed with dynamic mesh with smoothing and the solution was initialized with hybrid initialization. Hence, volumetric plots and the net flow rates were obtained at different frequencies. The volumetric plot of velocity profile at the compression phase and the variation of net flow rates with frequency are illustrated in Figure 5 and Figure 6 respectively.

## 4. Fabrication of Micropump

The proposed design was fabricated with polymethyl methacrylate (PMMA) plates. The commercially available diaphragms bonded with PZT-5H discs were used as PZT actuators (http://www.murata.com). Thin layers of PMMA plates were prepared, then all the thin layers were bonded with proper binding technique. “Isopropyl alcohol” was used as a binder medium and then thermally treated to 68 °C to avoid leakages. This is the latest simple and fast thermally-solvent assisted technique for the bonding of PMMA–PMMA in microfluidics applications [26]. 

**Figure 7:**
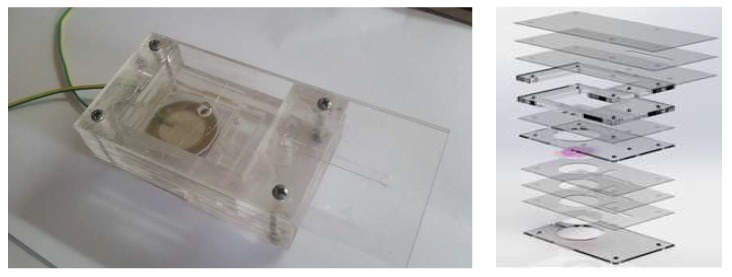
Top and exploded views fabricated micropump.

The fabricated micropump contained a pump chamber, reservoir, and testing plate. There were three 1 mm plates used to make the pump chamber and the chamber was enclosed with two piezoelectric transducers. The rest of the PMMA plates were used to fabricate the reservoir as well as for the testing requirements. The capacity of the reservoir was 10 mL. The top view of the fabricated micropump and its exploded view is presented in Figure 7 respectively. 

## 5. Signal Conditioning Circuit Design

**Figure 8:**
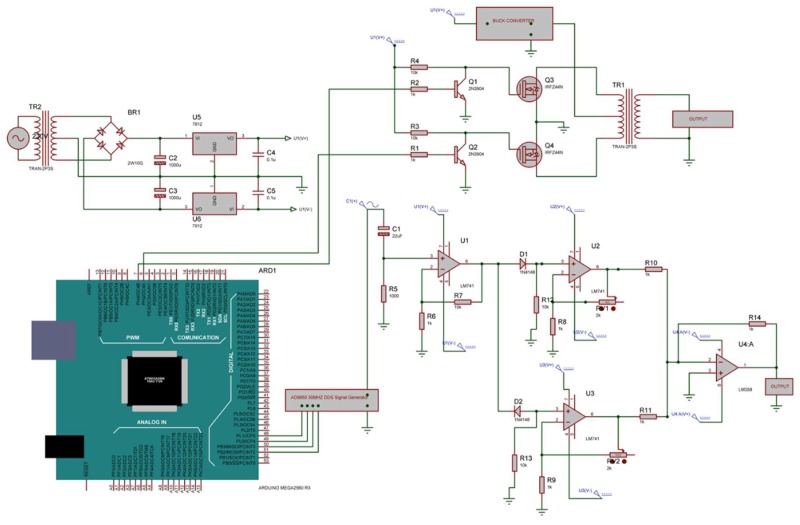
The overall design of the signal conditioning circuit.

The micropump was controlled by a signal generator and the frequency of this was controlled by an Arduino mega 256 controller. To control PZT diaphragms using sine waves, an AD9850 Signal Generator Module with a simple designed circuit was used since this can create a pure frequency and phase programmable, analog sine waves relative to an accurate clock source. The AC sine wave was amplified using an LM741 operational amplifier in the first stage of the amplifier circuit since operational amplifiers are stable, easy to use, and they are ideal for both long-term and short-term uses. Also, they are readily varied and adjusted by working on the Av (gain) value. Figure 8 shows the overall design of the signal conditioning circuit. 

## 6. Characterization of the Micropump

**Figure 9:**
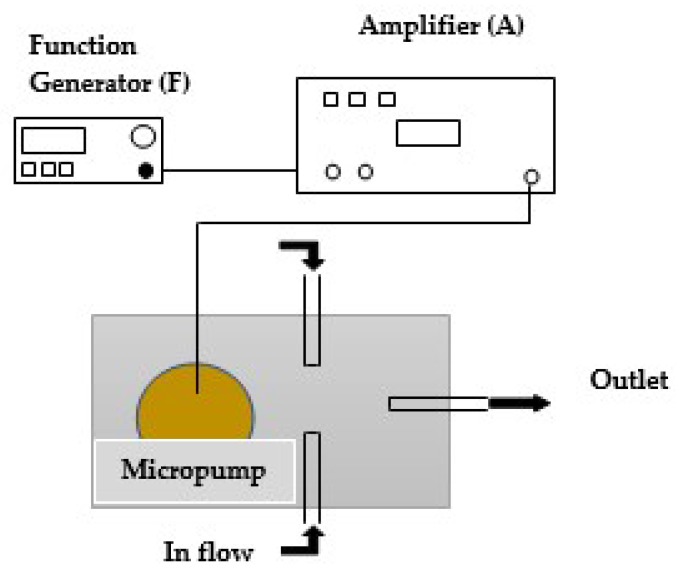
The schematic illustration of Experimental setup.

The developed experimental setup of the micropump is illustrated in Figure 9. According to this experimental setup, the time taken to collect 5 mL of water through the outlet was measured by stopwatch. The discharge head was kept at 0 mm and the frequency was varied. Hence, the net flow rates at different frequencies were obtained. In addition 5 mm of clear acrylic were added plate-by-plate to the micropump in order to obtain experimental data sets for different discharged heads. Then, the time taken was measured to fill the 5 mL at 50 Hz frequency and the discharged head at different time intervals were observed.

## 7. Results and Discussion

**Figure 10:**
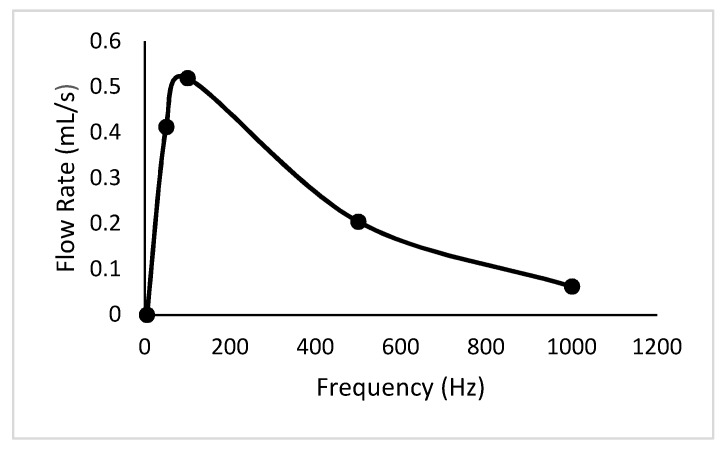
Variation of net flow rates with frequency.

**Figure 11:**
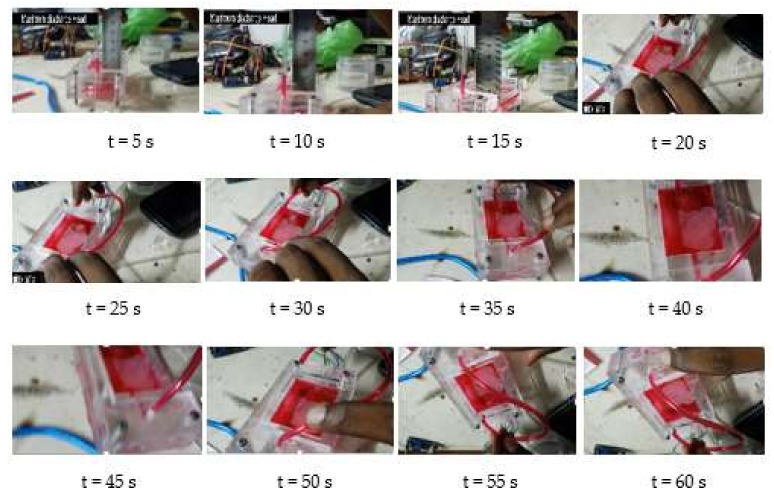
Flow rate of the fabricated pump through one minute.

**Figure 12:**
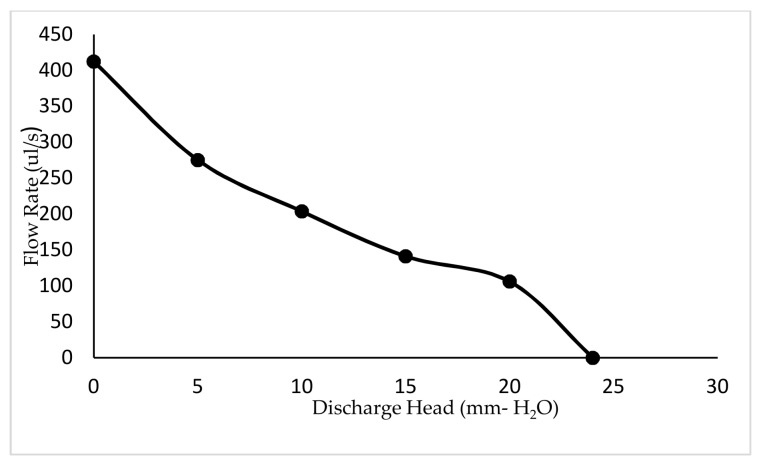
Variation of net flow rates with discharged head, the driving voltage on PZT diaphragm is 20 V.

The variation of net flow rates with frequency is plotted in Figure 10. In addition, the states at every five seconds through one minute, as well as the variation of flow rates with discharged heads, are expressed in Figure 11 and Figure 12 respectively. According to the respective plots, the net flow rate rapidly increased from 0 Hz to about 100 Hz and then tended to 0. This happened due to a partial contribution of eigenfrequency of the piezoelectric diaphragm and the resonant frequency of the fluid–solid interaction. In addition, the increment in discharged head gave smaller flow rates, which is a mandatory requirement to identify the flow characteristics when the pump is designed to a specified application. 

## 8. Conclusions and Future Direction

A piezoelectrically actuated valveless micropump was designed and developed with the specified control circuit. From the test results, it is interpreted that by increasing the frequency from 0 Hz to a specific value, the flow rate increases and after that, the flow rate decreases. In addition, the numerical simulation carried out to validate the test results revealed the same behavior. According to the test results, the maximum net flow rate was 512 µL/s at 100 Hz. The developed pump was tested for a constant applied frequency and a different discharge head pressure. The test results showed that the flow rate decreases with the increasing head, and this gives the maximum pump head of 24 mm H_2_O respectively. This pump curve for the developed pump is essential to identify the flow characteristics when the pump is designed to a specified application. Thus, piezoelectric and microfluidic simulation analysis delivered essential information relating to operating frequency, working principle, and the flow rate of the device. Though micropumps have many applications, this design is mainly focused on the field of biomedical applications. Material selection for this micropump should be reviewed in order to select the compatible material for the specific application in biomedicine. Hence, further studies will also be carried out to miniaturize the device to the micro scale.

## Figures and Tables

**Table 1:** Material properties of brass.

Description	Value
Density (kg/m^3^)	8360
Young’s modulus (GPa)	125
Poisson’s ratio	0.33

## References

[1] Laser D.J., Santiago J.G. (2004). A review of micropumps. J. Micromech. Microeng..

[2] Munas F.R., Amarasinghe Y.W.R., Dao D. (2015). Review on MEMS based Micropumps for Biomedical Applications. Int. J. Innov. Res. Sci. Eng. Technol..

[3] Nisar A., Afzulpurkar N., Mahaisavariya B., Tuantranont A. (2008). MEMS-based micropumps in drug delivery and biomedical applications. Sens. Actuators B Chem..

[4] Yang H., Tsai T.H., Hu C.C. Portable Valve-less Peristaltic Micro Pump Design and Fabrication. Proceedings of the 2008 IEEE Symposium on Design, Test, Integration and Packaging of MEMS/MOEMS.

[5] Shukur A.F.M., Sabani N., Taib B.N., Azidin M.A.M., Shahimin M.M. (2013). Performance characteristics of valveless and cantilever-valve micropump. Proc. SPIE.

[6] Feng G.H., Kim E.S. (2005). Piezoelectrically Actuated Dome-shaped Diaphragm Micropump. IEEE J. Microelectromech. Syst..

[7] Abhari F., Jaafar H., Yunus N.A. (2012). A Comprehensive Study of Micropumps Technologies. Int. J. Electrochem. Sci..

[8] Zengerle R., Ulrich J., Kluge S., Richter M., Richter A. (1995). A bidirectional silicon micropump. Sens. Actuators.

[9] Podder P.K., Mallick D., Samajdar D.P., Bhattacharyya A. Design, Simulation and Study of MEMS Based Micro-needles and Micro-pump for Biomedical Applications. Proceedings of the 2011 COMSOL Conference.

[10] Pandey M., Upadhyay P.C. (2012). Design and Simulation of Valveless PZT Micropump for Drug Delivery system. Int. J. Adv. Technol..

[11] Li S., Chen S. (2003). Analytical Analysis of a circular PZT actuator for valveless micropumps. Sens. Actuators A.

[12] Woias P. (2005). Micro Pumps—Past, progress and future prospects. Sens. Actuators B Chem..

[13] Tay F.E.H. (2002). Microfluidics and BioMEMS Applications.

[14] Spencer W.J., Corbett W.T., Dominguez L.R., Shafer B.D. (1978). An electronically controlled piezoelectric insulin pump and valves. IEEE Trans. Sonics Ultrason..

[15] Mishra R., Bhattacharyya T.K., Maiti T.K. Design and simulation of microfluidic components towards development of a controlled drug delivery platform. Proceedings of the 2016 IEEE International Conference on VLSI Design.

[16] Rojas J.J., Morales J.E. Design and Simulation of a Piezoelectric Actuated Valveless Micropump. Proceedings of the COMSOL Conference.

[17] Dau V.T., Dinh T.X., Sakamoto R., Tomonori O., Tanaka K., Sugiyama S. A Valve-less micropump with PZT diaphragm. Proceedings of the Twelfth International Conference on Miniaturized Systems for Chemistry and Life Sciences.

[18] Dau V.T., Dinh T.X., Sugiyama S. (2009). A MEMS-based silicon micropump with intersecting channels and integrated hotwires. J. Micromech. Microeng..

[19] Dau V.T., Dinh T.X., Ktsuhiko T., Sugiyama S. (2009). A cross-junction channel valve-less micropump with PZT Actuation. Microsyst. Technol..

[20] Dau V.T., Dinh T.X., Nguyen Q.D., Amarasinghe R., Tanaka K., Sugiyama S. Microfluidic valveless pump actuated by electromagnetic force. Proceedings of the IEEE Sensors.

[21] Dinh T.X., Le N.T.M., Dau V.T., Ogami Y. (2011). A dynamic model for studying valveless electromagnetic micropumps. J. Micromech. Microeng..

[22] Dich N.Q., Dinh T.X., Pham P.H., Dau V.T. (2015). Study of valveless electromagnetic micropump by volume-of-fluid and OpenFOAM. Jpn. J. Appl. Phys..

[23] Versteeg H.K., Malalasekera W. (1995). An Introduction to Computational Fluid Dynamics—The Finite Volume Method.

[24] Farshid. M., Ghaus R. Farshid. M.; Ghaus, R. Design and Simulation of MEMS Based Piezoelectric Insulin Micropump. Proceedings of the COMSOL Conference.

[25] Timoshenko S.P., Goodie J.N. (1970). Theory of Elasticity.

[26] Bamshad A., Nikfarjam A., Khaleghi H. (2016). A new simple and fast thermally solvent assisted method to bond PMMA–PMMA in micro-fluidics devices. J. Micromech. Microeng..

